# Marked increase in incidence for bloodstream infections due to *Escherichia coli*, a side effect of previous antibiotic therapy in the elderly

**DOI:** 10.3389/fmicb.2015.00646

**Published:** 2015-06-30

**Authors:** Nathalie L. van der Mee-Marquet, Dominique S. Blanc, Houssein Gbaguidi-Haore, Sandra Dos Santos Borges, Quentin Viboud, Xavier Bertrand, Roland Quentin

**Affiliations:** ^1^Service de Bactériologie et Hygiène, Centre Hospitalier Universitaire de Tours, UMR 1282Tours, France; ^2^Réseau des Hygiénistes du Centre, Centre Hospitalier Universitaire de ToursFrance; ^3^Service of Hospital Preventive Medicine, Lausanne University HospitalLausanne, Switzerland; ^4^Service d'Hygiéne Hospitaliére, Centre Hospitalier Universitaire de Besançon, UMR CNRS 6249, Chrono-environnement, Université Bourgogne Framche-ComtéBesançon, France

**Keywords:** *Escherichia coli*, bloodstream infection, elderly, antibiotic, ST 131

## Abstract

We conducted a survey including 3334 bloodstream infections (BSIs) due to *E. coli* diagnosed in 2005–2014 at a stable cohort of hospitals. Marked increases in incidence were observed for community-acquired (CA) BSIs in patients aged >75 years, CA-BSIs of digestive origin in patients aged 60–74 years, healthcare-associated BSIs, and BSIs associated with ESBL (extended-spectrum B-lactamase)-producing *E. coli* (ESBLEc). Using MLST, we studied the genetic diversity of 412 BSI isolates recovered during the 2014 survey: 7 major sequence type complexes (STCs) were revealed in phylogenetic group B2, 3 in group A/B1 and 2 in group D. Among the 31 ESBLEc isolates, 1/3 belonged to STC 131. We searched for possible associations between clonal groups, clinical determinants and characteristics of BSIs: isolates from groups B2 (except STC 131) and D were susceptible to antibiotics and associated with BSIs of urinary origin in patients <60 years. STC 131 and group A/B1 isolates were multi-drug resistant and associated with CA-BSIs of digestive origin in patients aged 60–74 with a recent history of antibiotic treatment. STC 131 isolates were associated with HCA-BSIs in patients with recent/present hospitalization in a long-stay unit. We provide a unique population-based picture of the epidemiology of *E. coli* BSI. The aging nature of the population led to an increase in the number of cases caused by the B2 and D isolates generally implicated in BSIs. In addition, the association of a trend toward increasing rates of gut colonization with multi drug-resistant isolates revealed by the rise in the incidence of BSIs of digestive origin caused by STC 131 and A/B1 (STCs 10, 23, and 155) isolates, and a significant increase in the frequency of BSIs in elderly patients with recent antibiotic treatment suggested that antibiotic use may have contributed to the growing incidence of BSI.

## Introduction

Bloodstream infections (BSIs) are among the most severe human infections, with markedly high rates of morbidity and mortality. *E. coli* is the principal agent responsible for BSI in both patients with no recent history of healthcare (i.e., community-acquired, CA) (Laupland, 2013) and in patients recently or currently managed in a healthcare institution (i.e., healthcare-associated, HCA). *Escherichia coli* is a pathogen and a commensal organism of the mammalian intestine, usually described as “commensal,” “intestinal pathogenic,” or “extraintestinal pathogenic.” Intestinal pathogenic isolates cause diarrhea, mostly by producing toxins, and by invading and damaging the intestinal mucosa. Extraintestinal pathogenic isolates (ExPEC) cause urinary tract infections and BSIs through the production of toxins, adhesins, polysaccharide capsules, siderophores, and invasins, enabling them to evade host defenses and invade host tissues. Phylogenetic analyses have assigned *E. coli* isolates to six phylogenetic groups (A, B1, B2, C, D, and E). ExPEC strains generally belong to group B2, and, to a lesser extent, group D, whereas commensal strains and less virulent strains belong to group A or B1 (Tenaillon et al., 2010).

Various networks recently reported changes in the epidemiological features of *E. coli* infections in humans, showing an increase in the incidence of BSIs (Laupland, 2013; Williamson et al., 2013; Lai et al., 2014). Molecular studies showed that five major lineages (ST complexes 31, 69, 73, 95, and 131) were responsible for most ExPEC infections in humans and food animals (Maluta et al., 2014; Riley, 2014). Matches between isolates recovered from food animals and clinical isolates have suggested that the food animal reservoir may have contributed to the observed epidemiological changes in human infections (Manges et al., 2001; Vincent et al., 2010; Lazarus et al., 2015).

The infection control network of the Centre region of France annually conducts a prospective longitudinal BSI survey, to investigate the overall incidence of BSIs in the population and to improve our understanding of the course of these infections. In this work, we examined regional trends in the incidence of particular subgroups of BSIs (i.e., CA- and HCA-BSIs, BSIs of digestive or urinary origin, and BSIs associated with ESBL-producing isolates). The genetic characteristics of the *E. coli* BSI isolates were investigated, by characterizing isolates associated with BSI cases identified during the 2014 survey and searching for possible associations between clonal groups, clinical determinants and characteristics of BSIs.

## Materials and methods

### BSI epidemiological survey

The Centre region of France had a population of approximately 2.6 million at the time of the study (Supplementary Table 1). An annual 3-month survey of all cases of BSIs has been conducted in this region since 2000 (van der Mee-Marquet et al., 2004). In 2005–2014, a stable cohort of 32 hospitals participated in this program. These institutions had a total of 6904 short-stay beds and 3308 long-stay beds, accounting for 81.9% of the short-stay and 59.5% of the long-stay beds in the entire region. The survey covered 7,055,503 patient-days (PDs). The methods and study design have been reported elsewhere (van der Mee-Marquet et al., 2004). Briefly, BSI was defined as a positive blood culture from a patient with clinical or laboratory evidence of infection. The variables reported included patient age and sex, origin of the BSI [skin (primary cutaneous form or superinfection of a skin wound), surgical site, lungs, urinary tract, intravascular device, intra-abdominal or digestive tract], CA- or HCA-BSI and death within 7 days of BSI diagnosis. The origin of the BSI was determined by the doctor responsible for patient care, taking clinical and biological data into account. During the 2014 survey period, incontinence, recent history of hospitalization, living in a nursing home, and recent antibiotic treatment were also recorded. Data were analyzed with Epi Info version 6 software. Ethics approval for the survey was obtained from the *Réseau Alerte Investigation Surveillance des Infections Nosocomiales* (RAISIN). The study was managed jointly with the directors of the hospitals, the doctors responsible for patient care and the regional infection-control team.

### Microbiological methods

#### Bacteriology and antimicrobial susceptibility testing

During the 2005–2014 survey period, microbial identification and antimicrobial drug susceptibility testing were performed in the laboratories of the 32 participating centers. These laboratories used procedures in accordance with the recommendations of the French Antibiogram Committee and were subjected to external quality control (van der Mee-Marquet et al., 2003, http://www.sfm-microbiologie.org). For each case of BSI due to *E. coli*, the susceptibilities to ampicillin and cefotaxime of the corresponding isolate were reported, together with the clinical data. All isolates showing decreased susceptibility to second/third-generation cephalosporins were tested for ESBL production. A reduced susceptibility to cephalosporins was defined according to the French guidelines (http://www.sfm-microbiologie.org). In the case of a diminished susceptibility of the isolate (MIC > 1 mg/L) for at least one of the six cephalosporins (cefotaxime, ceftriaxone, ceftazidime, cefepime, cefpirome, cefixime), the production of ESBL was searched by the double-disk synergy test.

In 2014, all *E. coli* isolates from BSI cases were sent to the central laboratory for extensive study. *E. coli* identification was confirmed by MALDI-TOF MS (BioMérieux, Lyon, France). Antimicrobial drug susceptibility testing was performed by the agar disk diffusion method (http://www.sfm-microbiologie.org), and isolates displaying decreased susceptibility to second/third-generation cephalosporins were tested for ESBL production.

PCR assays were used to characterize the molecular mechanism associated with ESBL production, as described by Brechet et al. (2014).

MLST was used to study the genetic diversity of the isolates, as described by Wirth et al. (2006). All the allelic profiles obtained were analyzed, sequence type (ST) and ST complex (STC) were assigned according to the *E. coli* MLST database (http://mlst.warwick.ac.uk/mlst/). STCs were defined as groups of at least 3 STs sharing 6 alleles in pair-wise comparisons. Previous publications and the eBURST algorithm were used to assign STCs to STs that were not available from the *E. coli* MLST database. A neighbor-joining tree of the seven concatenated MLST genes was also used to classify our collection of isolates. The analysis was performed with MEGA6 software.

#### Data analysis

We took fluctuations in the population of the region during the study period into account, by expressing the incidence of CA-BSI cases (numerator) relative to the population of the region (denominator). The population data were obtained from the INSEE national database (*Institut National de la Statistique et des Etudes Economiques*, http://www.insee.fr). As our network covered most of the hospital centers of the region, we were confident that the participating centers were representative of the whole region. The statistical significance of temporal trends was assessed by Poisson or negative binomial regression for incidence rates, the Cochran–Armitage test for proportions and Spearman's rank correlation analysis for continuous variables. For categorical variables, we used Pearson's chi-squared test to compare groups. All analyses were two-tailed and a *p* < 0.05 was considered significant. We used Stata version 10.0 software (Stata Corp., College Station, TX, USA) for statistical analysis.

## Results

### Epidemiology of *E. coli* BSIs over a decade

From 2005 to 2014, the overall population of the region increased by 2.8%. The population of elderly people aged between 60 and 74 increased by 23.4%, the very old (>74) by 16.8%, whereas the population of people aged between 20 and 39 years decreased by 9% (Supplementary Table 1). Over this period, 3334 cases of BSI due to *E. coli* were identified, 1483 in male patients and 1851 in female patients. Most of the patients were hospitalized in short-stay units (95.3%). CA-BSIs accounted for 2209 cases (Table 1), whereas 1125 were attributed to HA-BSIs (Table 2).

**Table 1:** **Demographic and clinical characteristics of 2209 CA-BSIs**.

	**2005**	**2006**	**2007**	**2008**	**2009**	**2010**	**2011**	**2012**	**2013**	**2014**	**All**
**PATIENTS**	**108**	**187**	**221**	**213**	**249**	**231**	**235**	**231**	**250**	**284**	**2209**
Male/female	41/67	72/115	89/132	92/121	99/150	85/146	98/137	87/144	119/131	112/172	894/1315
M/F ratio	0.6	0.6	0.7	0.8	0.7	0.6	0.7	0.6	0.9	0.6	0.7
**Patient's age (mean/median)**											
Male	71/73	68/71	69/74	69/74	70/76	71/74	74/76	73/76	70/76	74/78	71/75
Female	70/74	63/72	66/75	68/77	70/77	70/77	72/78	70/78	74/74	72/80	70/77
Age group < 60	23	55	56	53	51	56	38	46	49	55	466
60–74	35	55	55	52	61	50	59	59	54	60	540
≥75	50	77	110	108	137	125	138	126	147	169	1187
Death within 7 days (%)	10 (9.3)	14 (7.5)	14 (6.3)	21 (9.9)	15 (6.0)	7 (3.0)	11 (4.7)	13 (5.6)	14 (5.6)	16 (5.6)	135 (6.1)
**BSI**											
Urinary origin (%)	67 (62.0)	125 (66.8)	132 (59.7)	122 (57.3)	156 (62.6)	121 (52.4)	141 (60.0)	135 (58.4)	141 (56.4)	165 (58.1)	1305 (59.1)
Male	18	41	41	37	41	29	46	31	46	41	371
Female	49	84	91	85	115	92	95	104	95	124	934
Age group < 60	16	39	35	32	38	35	29	33	25	41	323
60–74	21	39	37	24	36	22	35	32	28	30	304
≥75	30	47	60	66	82	64	77	70	88	94	678
Digestive origin (%)	23 (21.3)	38 (20.3)	55 (24.9)	41 (19.2)	47 (18.9)	70 (30.3)	57 (24.2)	61 (26.4)	78 (31.2)	84 (29.6)	556 (25.2)
Male	11	18	29	24	31	38	29	36	57	48	321
Age group < 60	1	4	5	3	5	9	4	5	13	9	58
60–74	5	7	8	10	5	12	10	15	17	14	103
≥75	5	7	16	11	21	17	15	16	27	25	160
Female	12	20	26	17	16	32	28	25	23	36	235
Age group < 60	2	5	4	2		4	2	3	5	1	28
60–74	2	3	5	3	3	6	5	3	7	10	47
≥75	8	12	17	12	13	22	21	19	11	25	160
Pulmonary origin	5	1	5	7	3	5	7	9	5	9	56
Other origin	4	8	8	14	13	11	12	6	14	3	93
Origin unknown	9	15	21	29	30	24	18	20	10	23	199

**Table 2:** **Demographic and clinical characteristics of 1125 HCA-BSIs**.

	**2005**	**2006**	**2007**	**2008**	**2009**	**2010**	**2011**	**2012**	**2013**	**2014**	**All**
Patient days in HCIs	370,250	444,518	456,053	486,226	492,269	446,157	438,624	483,757	470,028	474,953	4,562,835
Patients	58	91	112	110	108	121	112	120	134	159	1125
Male/female	27/31	45/46	56/56	69/41	59/49	65/56	51/61	66/54	70/64	81/78	589/536
M/F ratio	0.9	1.0	1.0	1.7	1.2	1.2	0.8	1.2	1.1	1.0	1.1
**Patient's age (mean/median)**											
Male	74/77	67/71	69/71	74/76	73/75	67/70	68/76	72/75	72/74	71/73	74/77
Female	72/75	71/79	74/76	72/80	72/76	69/75	71/77	74/79	77/78	70/75	72/77
Age group < 60	12	18	23	24	14	24	27	20	19	32	213
60–74	18	21	36	31	36	36	37	31	37	49	332
≥75	28	52	53	55	58	61	48	69	78	78	580
**Recent**											
Urinary catheterization (%)	18 (31.0)	23 (25.3)	26 (23.2)	29 (26.4)	41 (38.0)	33 (27.3)	26 (23.2)	40 (33.3)	34 (25.4)	56 (35.2)	326 (29.0)
Mechanical ventilation (%)		1 (1.1)		1 (0.9)	3 (2.8)	1 (0.8)	2 (1.8)	2 (1.7)		4 (2.5)	14 (1.2)
Death within 7 days (%)	5 (8.6)	9 (9.9)	10 (8.9)	16 (14.5)	13 (12.0)	18 (14.9)	10 (8.9)	12 (10.0)	17 (12.7)	13 (8.2)	123 (10.9)
**BSI**											
Urinary origin (%)	35 (60.3)	55 (60.4)	58 (51.8)	57 (51.8)	65 (60.2)	58 (47.9)	57 (50.9)	70 (58.3)	67 (50.0)	88 (55.3)	610 (54.2)
Digestive origin (%)	10 (17.2)	10 (11.0)	22 (19.6)	20 (18.2)	14 (13.0)	22 (18.2)	14 (12.5)	20 (16.7)	31 (23.1)	26 (16.3)	189 (16.8)
Pulmonary origin				2	4	5	5	3	4	7	30
Other origin	11	11	25	20	12	26	29	18	27	22	201
Origin unknown	2	15	7	11	13	10	7	9	5	16	95

#### Community-acquired BSIs

**Figure 1:**
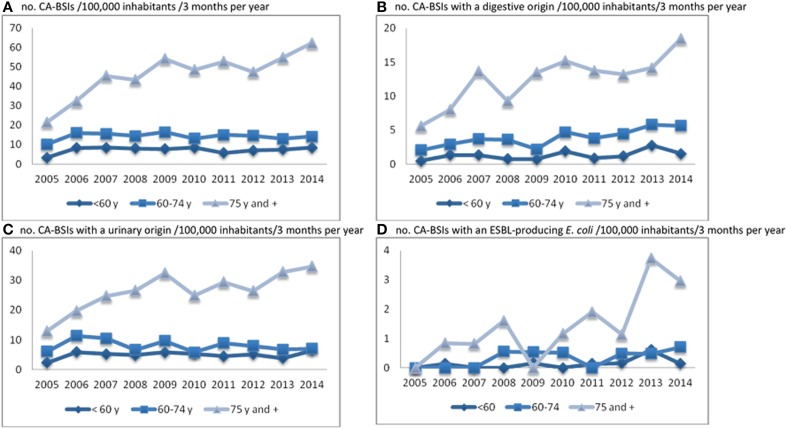
**Trends in CA-BSI incidence (no. of cases/100,000 inhabitants/3 months per year) by age group (A), by origin of BSI (B and C) and for BSIs with ESBL-producing** ***E. coli*** **isolates (D)**.

A major increase in the incidence (+156%, significant upward trend, *p* < 0.001) of CA-BSI was observed (number of cases per 100,000 inhabitants/3 months of a year), with similar trends observed for male (+166%) and female (+149%) patients. This global trend was almost entirely due to a significant increase in the incidence of BSI in people >74 years old (*p* < 0.001), with no major change observed in the other age groups (Figure 1A). Consistently, the median age of the patients increased significantly during the study period, from 73 to 78 years for men (*p* < 0.001), and from 74 to 80 for women (*p* = 0.035) (Table 1). There was a trend toward an increase in the incidence of BSIs of digestive (Figure 1B) or urinary origin (Figure 1C). This trend was similar for male and female patients for urinary origin (c.a. 170%), but differed significantly between the sexes for digestive origin (male, 319%, *p* < 0.001; female, 171%, *p* = 0.002); Table 1). A major increase in the incidence of BSIs associated with an ESBLEc was observed (+291%; *p* < 0.001; Table 3 and Figure 1D), mostly in the very old (Figure 1D). Note that the only trend toward an increase in patients <74 years concerned BSIs of digestive origin in patients aged 60–74 years (+177%; *p* = 0.001; Figure 1B). The early mortality rate did not increase significantly during the study period.

**Table 3:** **Antimicrobial susceptibility patterns of *E. coli* isolates associated with CA- and HCA-BSIs**.

**Antimicrobial susceptibility patterns of *E. coli* isolates**	**2005**	**2006**	**2007**	**2008**	**2009**	**2010**	**2011**	**2012**	**2013**	**2014**	**All (%)**
Amoxicillin S	81	137	140	147	175	182	175	158	163	201	1559 (46.8)
In CA-BSIs	55	97	97	98	128	129	133	115	112	135	1099 (49.7)
In HCA-BSIs	26	40	43	49	47	53	42	43	51	66	460 (40.9)
Amoxicillin R cefotaxime S	77	129	175	153	149	146	153	157	185	198	1522 (45.6)
In CA-BSIs	49	84	114	100	98	89	92	98	115	128	967 (43.8)
In HCA-BSIs	28	45	61	53	51	57	61	59	70	70	555 (49.3)
Amoxicillin R cefotaxime R ESBL+	1	5	7	11	8	14	14	20	26	32	138 (4.1)
In CA-BSIs		3	2	6	3	5	6	8	16	12	61 (2.8)
In HCA-BSIs	1	2	5	5	5	9	8	12	10	20	77 (6.8)
Amoxicillin R cefotaxime R (no ESBL)	3	2	2	1	5	5	4	13	9	5	49 (1.5)
In CA-BSIs	1			1	3	3	3	9	6	4	30 (1.4)
In HCA-BSIs	2	2	2		2	2	1	4	3	1	19 (1.7)
Not known	4	5	9	11	20	5	1	3	1	7	66 (2.0)
All (CA-BSIs/HCA-BSIs)	166 (108/58)	278 (187/91)	333 (221/112)	323 (213/110)	357 (249/108)	352 (231/121)	347 (235/112)	351 (231/120)	384 (250/134)	443 (284/159)	3334 (2209/1125)

#### Healthcare-associated (HA) BSIs

**Figure 2:**
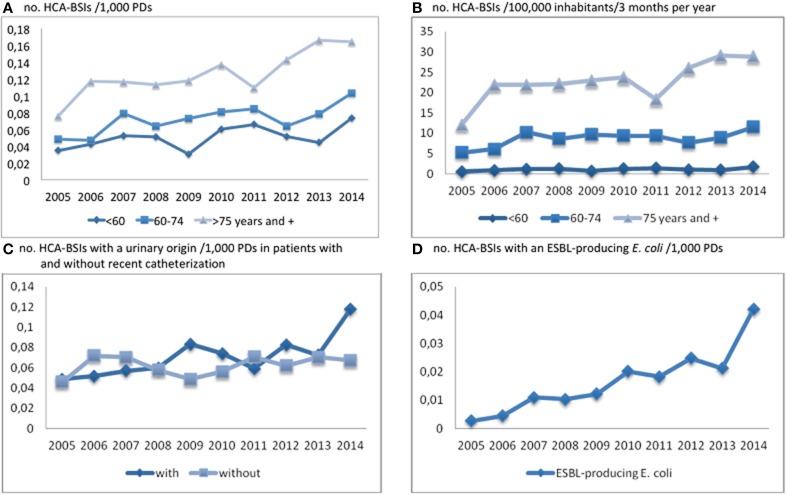
**Trends in HCA-BSI incidence (no. of cases/1000 PDs and/100,000 inhabitants/3 months per year) by age group (A and B), for BSIs with a urinary origin in patients with and without recent catheterization (C) and for BSIs with ESBL-producing** ***E. coli*** **isolates (D)**.

A 114% increase in incidence was identified for HCA-BSIs (number of cases per 1000 PDs, Table 2, Figure 2A). This global trend was due to BSIs in patients >74 years old, but also, to a lesser extent, patients aged 60–74 years (Figure 2B). By contrast with CA-BSI cases, the median age of patients with HCA-BSI did not change during the study period (Table 2). The observed increases were similar for male and female patients, and mostly concerned BSIs of urinary origin (+96%). The increasing incidence of BSI cases with a urinary origin was similar for patients with and without a recent history of urinary catheterization (Figure 2C). A large increase was observed in the incidence of BSIs associated with ESBLEc (0.003/1000 PDs in 2005 and 0.042 in 2014; *p* < 0.001) (Table 3 and Figure 2D). As for CA-BSI, the early mortality rate did not increase significantly during the study period.

### Molecular characterization of *E. coli* isolates

In total, 412 (93.0%) isolates were available for molecular analysis, from the 443 BSIs diagnosed in 2014. The clinical determinants and characteristics of these 412 BSIs are presented in Table 4.

**Table 4:** **Clinical data, characteristics of 412 BSIs and molecular characteristics of the related *E. coli* isolates**.

	**B2**		**D**	**A/B1**	**Others**	**All**
	**ST131**	**Other B2**				
**PATIENTS**	**25**	**204**	**56**	**106**	**21**	**412**
Sex (male/female) (ratio)	14/11 (1.3)	91/113 (0.8)	19/37(0.5)	48/58 (0.8)	14/7 (2.0)	186/226 (0.8)
Age mean/median	70/71	72/80	73/80	75/78	73/76	73/78
Age group < 60 (%)	6 (24.0)	44 (21.6)	13 (23.2)	9 (8.5)	4 (19.0)	76 (18.5)
60–74 (%)	8 (32.0)	40 (19.6)	9 (16.1)	38 (35.8)	4 (19.0)	99 (24.0)
≥75 (%)	11 (44.0)	120 (58.8)	34 (60.7)	59 (55.7)	13 (61.9)	237 (57.5)
Incontinence U/F	8/5	43/23	11/7	16/4	2/1	80/40
**Recent**
Urinary catheterization (%)	9 (36.0)	27 (13.2)	6 (10.7)	8 (7.5)	2 (9.5)	52 (12.6)
Stay in RC/LSU (%)	5 (20.0)	9 (4.4)	2 (3.6)	5 (4.7)	1 (4.8)	22 (5.3)
Living in nursing home (%)	4 (16.0)	18 (8.8)	6 (10.7)	4 (3.8)	2 (9.5)	34 (8.2)
Antibiotic treatment (%)	13 (52.0)	41 (20.1)	16 (28.6)	38 (35.8)	7 (33.3)	115 (27.9)
Fluoroquinolone	2 (8.0)	3 (1.5)	3 (5.4)	4 (3.8)	1 (4.8)	13 (3.2)
Cephalosporin	3 (12.0)	11 (5.4)	5 (8.9)	14 (13.2)		33 (8.0)
Death within 7 days (%)		17 (8.3)	4 (7.1)	7 (6.6)		28 (6.8)
**BSI**						
CA/HCA (ratio)	11/14 (0.8)	133/71 (1.9)	34/22 (1.5)	68/38 (1.8)	12/9 (1.3)	258/154 (1.7)
Urinary source (%)	17 (68.0)	134 (65.7)	34 (60.7)	45 (42.4)	7 (33.3)	237 (57.5)
CA-BSI (%)	5 (45.4)	92 (69.2)	20 (58.8)	31 (45.6)	5 (23.8)	153 (59.3)
HCA-BSI (%)	12 (85.7)	42 (59.1)	14 (63.6)	14 (36.8)	2 (9.5)	84 (54.5)
Digestive source (%)	7 (28.0)	30 (14.7)	13 (23.2)	39 (36.8)	12 (57.1)	101 (25.5)
CA-BSI (%)	6 (54.5)	22 (16.5)	9 (26.5)	29 (42.6)	2 (9.5)	74 (28.7)
HCA-BSI (%)	1 (7.1)	8 (11.0)	4 (18.2)	10 (26.3)	1 (4.8)	27 (17.5)
Other sources/not known	1/0	18/22	5/4	11/11	0/2	35/39
***E. COLI* ISOLATES**						
**Antibiotic susceptibility pattern**						
Susceptible to antibiotic tested (%)	3 (12.0)	140 (68.6)	13 (23.2)	32 (30.2)	11 (52.4)	199 (48.3)
FQ R		1	1	2		4
SXT/TMP R				2		2
FQ + SXT/TMP R				1	2	3
SXT/TMP + To/Gn R				1		1
AMX R (%)	2 (8.0)	41 (20.1)	18 (32.1)	18 (17.0)		79 (19.2)
AMX + FQ R (%)	2 (8.0)	2 (1.0)	1 (1.8)	9 (8.5)		14 (3.4)
AMX + SXT/TMP R (%)		16 (7.8)	11 (19.6)	14 (13.2)	6 (28.6)	47 (11.4)
AMX + To/Gn R			2			2
AMX + FQ + SXT/TMP R			3	4	1 (4.8)	8
AMX + FQ + To/Gn R	3					3
AMX + SXT/TMP + To/Gn R	1		2	1		4
AMX + FQ + SXT/TMP + To/Gn R (%)	3 (12.0)		1 (1.8)	8 (7.5)		12 (2.9)
AMX + FQ + SXT/TMP R (ESBL+)	1					1
CFX R (ESBL+)		1	1			2
CFX + FQ R (ESBL+)	2	1		2		5
CFX + SXT/TMP R (ESBL+)	1			3		4
CFX + SXT/TMP + To/Gn R (ESBL+)		1	1			2
CFX + FQ + SXT/TMP R (ESBL+)				3		3
CFX + FQ + To/Gn R (ESBL+) (%)	4 (16.0)					4 (1.0)
CFX + FQ + SXT/TMP + To/Gn R (ESBL+)	3 (12.0)	1 (0.5)	1 (1.8)	4 (3.8)	1 (4.8)	10 (2.4)
CFX R (no ESBL)				1		1
CFX + FQ + To/Gn R (no ESBL)				1		1
CFX + FQ + SXT/TMP R (no ESBL)			1			1
**Antibiotic resistance**						
CFX (%)	10 (40.0)	4 (2.0)	4 (7.1)	14 (13.2)	1 (4.8)	33 (8.0)
FQ (%)	18 (72.0)	5 (2.4)	8 (14.3)	34 (32.1)	4 (19.0)	69 (16.7)
SXT/TMP (%)	9 (36.0)	18 (8.8)	20 (35.7)	41 (38.7)	10 (47.6)	98 (23.8)
ESBL production (%)	11 (44.0)	4 (2.0)	3 (5.4)	12 (11.3)	1 (4.8)	31 (7.5)

#### Genetic diversity of isolates

**Figure 3:**
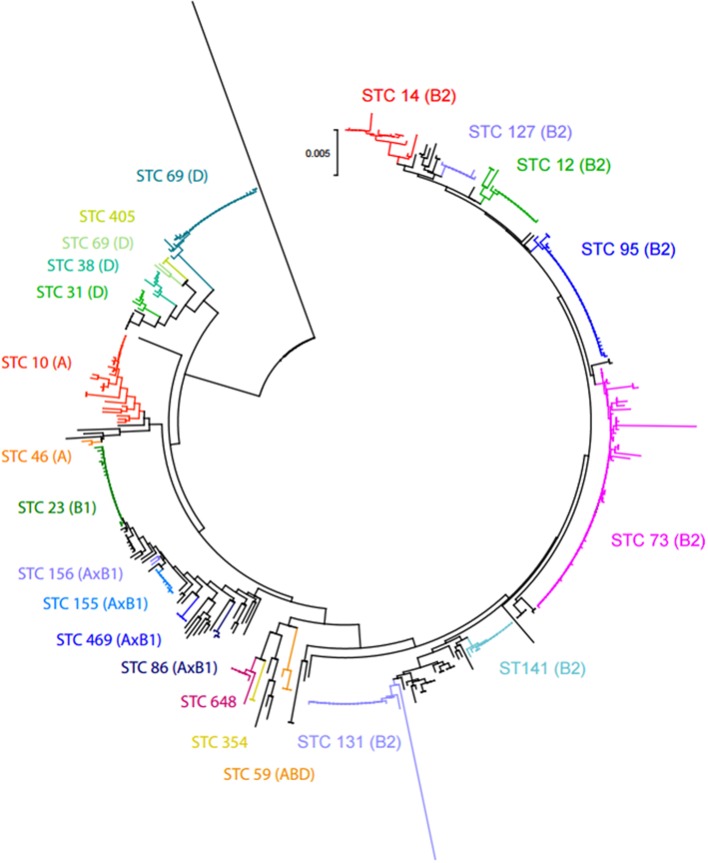
**Neighbor-joining tree obtained from the concatenated sequences of the 7 MLST loci of** ***E. coli*** **isolates from BSI in 2014**. The 12 major ST complexes (and their respective phylogroups) are placed on the tree.

MLST recovered 102 STs from the 412 isolates. The neighbor-joining tree obtained from the concatenated sequences revealed 12 major STCs containing 321 isolates (77.9%) (Figure 3). Seven of them (STCs 73, 95, 131, 14, 12, 141, and 92) belonged to group B2 and two to group D (STC 69 and 31). The three remaining major clones (STCs 23, 10, and 155) belonged to groups A and B1. The remaining 91 isolates belonged to unique STs or were placed in minor groups (Table 4). Overall, 55.6, 13.6, and 25.7% of the isolates studied belonged to groups B2, D, and A/B1, respectively.

#### Antimicrobial drug susceptibility

One hundred ninety-nine (48.3%) isolates were susceptible to all antibiotics tested. Seventy-nine were resistant to amoxicillin only (19.2%) and 47 (11.4%) were resistant to amoxicillin and trimethoprim/sulfamethozaxole (TMP/SXZ); 33 were resistant to cefotaxime (8.0%), all but two being ESBLEc (7.5%) (Table 4). In all but one case, the ESBL belonged to the CTX-M family. Most of the 30 CTX-M ESBLs were of types M15 (43.3%) and M1 (23.3%); 98 isolates were resistant to TMP/SXZ (23.8%), 69 to fluoroquinolone (16.7%) and 39 to tobramycin and gentamicin (9.5%). ESBLEc were mostly multidrug-resistant, with 74.2% resistant to fluoroquinolone, 64.5% to TMP/SXZ and 51.6% to tobramycin and gentamicin. None of the 412 isolates was resistant to imipenem or ertapenem.

#### Genotypes and clinical determinants

STC 131 isolates were associated with BSIs in patients hospitalized in a RC/LSU (*p* < 0.001), or recently hospitalized in a rehabilitation care-or long-stay unit (RC/LCU) (*p* = 0.007), and patients with recent urinary catheterization (*p* = 0.002) or recent antibiotic treatment (*p* < 0.006). Two characteristics were common to A/B1 and STC 131 isolates: the isolates from these groups were associated with BSIs in patients aged 60–74 years (*p* < 0.001) or recently treated with cephalosporin (*p* = 0.011).

#### Genotypes and the presumed origin of the BSI

A/B1 and STC 131 isolates were associated with CA-BSIs of digestive origin (*p* < 0.001); by contrast, B2 (except STC 131) and D isolates were associated with CA-BSIs with a urinary source (*p* = 0.005). STC 131 isolates were associated with HCA-BSIs (*p* = 0.047).

#### Genotypes and the antibiotic susceptibility of *E. coli* isolates

STC 131 isolates were associated with resistance to fluoroquinolones (*p* < 0.001). A/B1 and STC 131 isolates were associated with resistance to amoxicillin and fluoroquinolones (*p* < 0.001) and resistance to cefotaxime (*p* = 0.022). ESBL production was associated with isolates of STCs 10, 155, and 131 (*p* < 0.001). CTX-M15 ESBLs were associated with STC131 (*p* = 0.051) and CTX-M1 with STC 155 (*p* = 0.045). B2 isolates (except STC 131) were associated with susceptibility to all antibiotics tested (*p* < 0.001), and D isolates were associated with resistance to amoxicillin and TMP/SMZ (*p* = 0.016).

#### Clinical determinants, the characteristics of the BSIs and ESBLEc

ESBLEc-BSIs were associated with healthcare (*p* = 0.004), hospitalization in a LSU (*p* = 0.018), and recent antibiotic treatment (*p* = 0.002). ESBLEc were associated with resistance to fluoroquinolones, SXT/TMP and tobramycin/gentamicin (*p* < 0.001). The clinical determinants and characteristics of ESBLEc-BSIs were similar for all clonal groups other than STC 131, for which infection was associated with hospitalization in a long-stay unit (*p* = 0.042). For STC 131-BSIs, clinical determinants and the characteristics of the infection were similar for ESBLEc and non-ESBLEc.

## Discussion

This longitudinal prospective BSI survey provides a unique contemporary population-based picture of the epidemiology of *E. coli* BSI in a large part of France, and highlights two main factors that may have contributed to the growing incidence of BSIs due to *E. coli*: the aging of the population, and antibiotic use.

The increase in the incidence of BSIs due to *E. coli* observed in our population during the study period differed between age groups. The increase almost entirely concerned the very old (>74 years). Susceptibility to infectious diseases in the elderly is increased by a combination of factors, including immune senescence, denutrition, changes in skin and mucosal barrier functions, degenerative changes in bone and cartilage, a decrease in respiratory capacity and common comorbid conditions. BSI is a leading cause of morbidity and mortality in the geriatric population, and its frequency is higher in the elderly than in other age groups (Uslan et al., 2007). The subpopulation of individuals >74 years old increased over the study period in this region, consistent with the increase in the number of BSI cases.

However, the range of increases in BSI incidence was much larger than that for the very old subpopulation itself, suggesting a contribution of factors other than aging to the increase in BSI incidence. Regarding CA-BSIs only, an increase in BSI incidence was observed in patients aged 60–74 years, particularly for BSIs of digestive origin. The intestinal tract being the immediate reservoir of the ExPEC, these findings suggest that *E. coli* colonizing the gut may be increasingly able to cause BSI in the elderly.

CA- and HCA-BSIs, despite displaying an increase in incidence, did not lead to an increase in early mortality. This finding suggests that an increase in the virulence of the isolates involved in BSIs is unlikely. Nevertheless, this study had several limitations, including the lack of investigation of possible changes in therapeutic management by the physicians during the study, and the impossibility of analyzing BSIs from critically ill and moderately ill patients separately. Therefore, further studies should be conducted to clarify this point.

Consistent with recent reports for *E. coli* BSI isolates (Adams-Sapper et al., 2013; Burdet et al., 2014; Horner et al., 2014), most of the 412 studied isolates belonged to the major ExPEC, with a predominance of the B2 and D groups (STC 73, 95, 131 and 69). However, we found a lower proportion of BSIs due to group B2 and a higher proportion due to group A/B1 than in these previous reports (Jaureguy et al., 2008; Bert et al., 2010). We investigated BSI isolates from 2014, whereas previous reports investigated earlier isolates. Our results may indicate an increasing and recent implication of the A/B1 group in human BSIs.

Three major groups were defined on the basis of the characteristics of isolates and the clinical determinants of BSIs. First, isolates from groups B2 (except STC 131) and D were mostly highly susceptible to antibiotics and associated with BSIs of urinary origin in patients <60 years old, highlighting their well-recognized virulence. Second, STC 131, which included many isolates resistant to fluoroquinolones, and TMP/SXZ; they were ESBL-producers, and associated with HCA-BSIs of urinary origin in patients with recent/present hospitalization in long-stay units, and with CA-BSI of digestive origin in patients aged 60–74 years recently treated with antibiotics. The third group consisted of A/B1 isolates, usually considered commensal, with many features in common with STC 131 isolates regarding CA-BSIs. These isolates were frequently resistant to fluoroquinolones, TMP/SXZ and produced ESBL, and they were associated with CA-BSI cases of digestive origin in patients aged 60–74 years recently treated with antibiotics, particularly cephalosporins.

The A/B1 isolates of STCs 23, 10, and 155 have been shown to colonize food animals, to infect animals and humans (Manges et al., 2001; Huijbers et al., 2014; Maluta et al., 2014; Lazarus et al., 2015), and to be associated with perfect matches between isolates recovered from humans and chickens (Cortés et al., 2010; Kluytmans et al., 2013); they are thus considered to be associated with a zoonotic risk (Lazarus et al., 2015). Our study showed that BSI cases of digestive origin were mostly caused by isolates from these clonal complexes and STC 131. The number of BSI cases of digestive origin due to *E. coli* increased over the study period. Our results suggest that *E. coli* from the these clonal complexes probably colonize the intestines more frequently and/or successfully, thereby contributing to the growing number of BSIs observed over the study period.

The significant association of BSI with STC 131 and A/B1 isolates and recent antibiotic treatment suggested that prior exposure of the gut to antibiotics might have led to long-lasting, strong colonization by these isolates, this process being favored by the high frequency of antibiotic resistance genes in their genomes. Recent reports concerning antibiotic consumption (Bernier et al., 2014; http://www.cclin-arlin.fr, 2014)^1^ have described an increase in the use of antibiotics by the elderly over the study period. As data from our region were included in these studies, these trends toward an increase may apply to our region. Thus, our findings suggest that the increasing incidence of BSIs, often of digestive origin and mostly linked to antibiotic-resistant STC 131 and A/B1 isolates may at least partly result from the increasing use of antibiotics by inpatients and outpatients in our region during the study period.

Our last key finding was related to the large increase in the frequency of ESBLEc-BSIs. Prior antibiotic treatment was the only risk factor identified for these infections. We were unable to identify any characteristic linked to clonal group, other than for the CTX-M-15-ST131 clonal group, which was significantly more frequently identified in patients hospitalized in long-stay units. The key position of this lineage, probably due to its high capacity for intestine colonization and urinary tract infections (Vimont et al., 2012), highlights the need for infection control measures to limit the spread of this clone in units caring for elderly patients.

## Conclusion

This study has incidence data spanning 2005–2014 and provides insight into changes in the epidemiological features of BSIs due to *E. coli*. Nevertheless, molecular data were only obtained for the isolates recovered in 2014. Further studies should be performed in different geographic areas and with the incorporation of more clinical data, to improve our understanding of the dynamics of the increase in *E. coli* BSIs in the elderly population. Molecular studies should investigate the genetic factors associated with the ability of the group A/B1 isolates to cause increasing numbers of invasive infections. In addition, investigations are required to determine whether these antibiotic-resistant isolates colonizing the human gut originate from contaminated food products.

### Conflict of interest statement

The authors declare that the research was conducted in the absence of any commercial or financial relationships that could be construed as a potential conflict of interest.

## Footnotes

^1^Institut de Veille Sanitaire and Agence Nationale de Sécurité du Médicament et des Produits de santé. Consommation d'antibiotiques et résistance aux antibiotiques en France: nécessité d'une mobilisation determinée et durable. Available online at: http://www.cclin-arlin.fr/Campagnes/Antibiotiques/PLAQ_DMI_RATB-Ansm_BAT_14112014.pdf
