# Supplementary material for: Marked increase in incidence for bloodstream infections due to Escherichia coli, a side effect of previous antibiotic therapy in the elderly
Source: Front Microbiol. 2015 Jun 30;6:646. doi: 10.3389/fmicb.2015.00646 (PMC4485226; doi:10.3389/fmicb.2015.00646)
Supplement: Supplementary file 1 [file Table1.DOCX]

**Supplementary table 1.**

Changes in the population of the Centre region of France over the last decade.

| **Study period** | **No of males by age group** | | | | | |
| --- | --- | --- | --- | --- | --- | --- |
|  | **<20 years** | **20-39** | **40-59** | **60-74** | **75 and +** | **Total** |
| 2005 | 312 463 | 313 390 | 343 685 | 161 969 | 88 022 | **1 219 529** |
| 2006 | 313 046 | 311 260 | 348 058 | 161 737 | 90 083 | **1 224 184** |
| 2007 | 312 587 | 309 220 | 347 977 | 166 247 | 91 980 | **1 228 011** |
| 2008 | 311 134 | 306 722 | 347 094 | 171 048 | 94 642 | **1 230 640** |
| 2009 | 312 362 | 303 438 | 344 921 | 176 631 | 96 454 | **1 233 806** |
| 2010 | 313 943 | 299 240 | 344 095 | 182 533 | 98 862 | **1 238 673** |
| 2011 | 314 707 | 295 460 | 343 153 | 188 014 | 100 724 | **1 242 058** |
| 2012 | 314 120 | 291 689 | 343 475 | 193 106 | 102 444 | **1 244 834** |
| 2013 | 314 980 | 287 461 | 343 701 | 198 202 | 103 903 | **1 248 247** |
| 2014 | 316 380 | 283 145 | 343 547 | 203 111 | 105 144 | **1 251 327** |
|  | **No of females by age group** | | | | | |
| 2005 | 299 643 | 312 344 | 349 879 | 182 141 | 143 710 | **1 287 717** |
| 2006 | 300 683 | 310 078 | 355 288 | 181 795 | 147 539 | **1 295 383** |
| 2007 | 299 260 | 308 974 | 355 158 | 185 215 | 150 301 | **1 298 908** |
| 2008 | 297 674 | 306 019 | 354 456 | 189 058 | 153 741 | **1 300 948** |
| 2009 | 297 520 | 303 324 | 353 210 | 194 643 | 156 087 | **1 304 784** |
| 2010 | 299 316 | 299 328 | 353 134 | 199 287 | 158 327 | **1 309 392** |
| 2011 | 299 922 | 296 989 | 352 326 | 204 848 | 160 692 | **1 314 777** |
| 2012 | 299 255 | 294 101 | 352 062 | 210 420 | 162 914 | **1 318 752** |
| 2013 | 300 454 | 289 858 | 351 718 | 216 540 | 164 305 | **1 322 875** |
| 2014 | 301 806 | 285 582 | 351 760 | 221 513 | 165 486 | **1 326 147** |
